# Chicago Pathological Society

**Published:** 1889-01

**Authors:** 


### CHICAGO PATHOLOGICAL SOCIETY.

*Stated Meeting December 10, 1888.*

DR. JOHN D. SKEER, IN THE CHAIR.

DR. D. W. GRAHAM reported a case of

ABNORMAL APPETITE IN AN INFANT.

He showed a box of gravel-stones which a baby a year and a half old had passed from its bowels at one time. There were more than a hundred, many being as large as a hickory-nut. So far as could be learned the child had swallowed them all the day before. The stones caused no symptoms whatever while in the alimentary canal. The child had a persistent inclination to eat stones, lime, mortar, plastering and like substances. It would secrete itself behind an article of furniture in the house and quietly pick off and eat the plastering till discovered; or in the yard it would in the same way pick the mortar from between the stones in the wall. This habit continued until the first teething was completed, since which there has been no such disposition. The only apparent departure from a normal condition of health during all this time was a waxy, semi-transparent appearance of the skin, especially of the face. There are several forms of depraved appetite given by systematic writers on the subject, this particular form —a desire to eat or swallow substances having no nutritive value—being called *pica.*

DR. GRAHAM also exhibited

A POCKET KNIFE WHICH A BABY EIGHT MONTHS OLD HAD SWALLOWED, and on the fifty-third day had passed *per rectum.* The knife was about three inches long. The bone-plates on the sides of the knife, as well as more than half of the two blades, had disappeared during the transit. The mother had given the knife to the child to play with. For some weeks laxatives were given occasionally. About the fiftieth day a swelling or lump was discovered by the parents in the right iliac region. They were advised to palpate the swelling several times a day. In two or three days the swelling disappeared, and the knife was passed in the condition shown. No symptoms were caused by the knife at any time. This case occurred in the practice of Dr. J. H. Graham, of Iowa.

DR. J. M. PATTON reported the case of a child, two and a half years old, that had swallowed a breast-pin, the head and its soldered attachment being about the size of a cherry. The mother was attracted by the child making a noise and efforts at swallowing. On examination the pin-rod was found wanting. No disturbance was produced, and the child was let alone. Two days thereafter it was passed from the bowels.

Dr. W. L. Copeland said with reference to foreign bodies being swallowed by children without producing any symptoms, he would say that some years ago a child was brought to him reported as having swallowed a nickel cent, which was much larger and thicker than an ordinary copper cent. The act was followed by slight irritation and coughing. The child had been examined by several physicians before it was brought to him, some of whom held that there was irritation of the stomach; others bronchitis. The case failed to yield to any kind of treatment. Shortly before this he had a case of stricture of the œsophagus, and this one was brought to him to have an œsophageal tube passed. He passed a tube of medium size without difficulty, but failed to find any obstruction. An examination of the stomach and lungs revealed nothing abnormal. The mother was instructed to bring the child back for a subsequent examination, and about a month elapsed before he again saw the case. The coughing had returned, in the meantime, giving the child a great deal of trouble. At the last examination he introduced a large tube into the stomach, and on withdrawing it the child coughed up the cent.

Dr. A. B. Strong said that cases in which foreign bodies had passed into the stomach should be let alone. Physic should not be given. The foreign body would come away after a while. He could recall several cases that had come under his observation, and one particularly, in which a child had swallowed a penny. He watched the case to see whether symptoms of stricture would develop, but the child finally vomited the cent and recovered without having manifested any symptoms whatever.

Dr. C. D. Wescott said that during his term of service as assistant physician to the Insane Hospital at Kankakee, a number of cases came under his notice. The most alarming one he had met with was that of a patient who ran to a window, broke the glass, pulled out some of the broken fragments, chewed and swallowed them. There was no doubt, he said, as to the patient chewing and swallowing the glass, for pieces were found in his mouth under the tongue. Nothing of a therapeutic nature was resorted to; and the patient had no symptoms.

Dr. J. J. M. Angear said that some physicians in cases of this kind wanted to give a child an emetic immediately. He would not do this, but the first thing to be done, in his judgment, is to have the child fed well with the coarsest kinds of food for the purpose of making as much fæcal matter as possible, so that the alimentary canal would be greatly distended, thus including the foreign substance in the fæcal mass; then after a sufficient time has elapsed for the fæces to accumulate to a considerable extent, he would give a cathartic. He would agree with Dr. Strong, however, that the better plan is to let nature take her course.

Dr. H. N. Moyer reported a case of

SUICIDE BY FIVE OUNCES OF PARIS GREEN.

The case was that of a man who, in a row, had fired from a window at his son-in-law; the latter in haste to get away had fallen backward just at the moment the pistol was discharged. The deceased then turned the pistol toward his own breast and inflicted the wound described. This attempt at suicide proving futile, he fled, and some days later, doubtless believing himself a murderer, he took the poison with fatal effect. This is one of the largest doses of arsenic ever taken, in all amounting to nearly five ounces.

Dr. Moyer also reported a case of

FRACTURE OF THE FIFTH CERVICAL VERTEBRA.

A man was found, lying in a depression some three feet in depth between the curbstone and side-walk, in an unconscious condition. The bottom of this depression was formed by loose dirt. Death took place some four hours afterward without return of consciousness. Rigor-mortis was well marked, and there were no external marks of injury or violence. On reflecting the scalp an irregular ecchymosis (about one and a half inches in diameter) was found on the vertex. The brain and other organs appeared to be perfectly normal. A slight inequality in the body of the fifth cervical vertebra was noticed, and when the head was grasped and tilted backward, a complete transverse fracture of the body of the fifth cervical vertebra was brought into view. The circumstances in this case pointed to the possibility of homicide, but the opinion was expressed that such an injury could hardly have been produced by homicidal violence, in the absence of more distinct external marks of injury. Further, that the fracture was probably caused by a fall from the side-walk down the slight depression where the patient was found.

Professor A. E. Hoadley mentioned a case of dislocation of the cervical vertebra. The patient, a boy ten years of age, fell from a tree and struck upon his head, the distance being some seven or eight feet, and from that time on he could not move his head. After complaining of a good deal of pain, and his head being in a fixed condition for about a week, he was brought to him for examination. He discovered a well-marked dislocation between the third and fourth cervical bodies. The boy perfectly recovered. At first he concluded it was a dislocation, but subsequent examination had led him to doubt that conclusion and to rather favor the theory of fracture, as there was a well-marked provisional callus. He would take particular pains to examine the case again and see if the provisional callus has been absorbed. There was no bruise noticeable on the boy.

Dr. Wescott had seen one case of fracture of the fourth cervical vertebra in a woman whose weight was about 120 pounds. She fell upon her head a distance of some 10 or 12 feet. There were no marks of external violence. There was, however, a slight flow of blood from both the ears and nose, death occurring almost immediately.

Dr. Skeer then read a paper entitled

A CASE OF OBSCURE DISEASE OF THE BRAIN.

Mr. B. F. L., a machinist, aged 54 years, five feet and eight inches in height, weight 185 pounds, was always considered a strong healthy man: born in New York; moved to Chicago in 1866. Never had any severe sickness; had a bronchial cough in 1876 which passed off in about six weeks. Last June he had a slight paralysis which came on imperceptibly and without shock. It was confined to his left leg. His face was drawn to one side for a day or two, and there was a slight impairment of speech. The paralysis of his leg passed away in about two weeks. The patient did not consider himself sick, but could not understand *why* he did not get well. In December, 1887, he took a journey to California and was absent three months. He did not enjoy the climate or scenery; was impatient to hasten from place to place. He had a voracious appetite—seemed to be hungry all the time. He had to be restrained as to the kind and quantity of his diet; was well nourished. He could not control his emotions; he would cry at common ordinary events, and sometimes cough inordinately at little things, and keep it up for a considerable time. This much of his history I have obtained from his wife and brother. I examined him for the first time about 1st of September last, when he walked to my office and home again, a distance of fourteen blocks. He had all the appearances of a man in good health, and did not complain of any pain or indisposition. There was, however, a very slight paralysis of the left leg and arm. I found on examination, moderate hypertrophy of the heart; no history of rheumatism; pulse about 90, full, hard and regular; temperature subnormal; tongue clear; appetite good—too good. Bowels were regular, and urine normal. My diagnosis was either a slight hæmorrhage or a tubercle, fibroma or carcinoma of slow growth, the brain accommodating itself to the pressure. I made an unfavorable prognosis. Ergot and bromide of sodium were prescribed. On the 20th of September he was down to his place of business, came home in the evening and ate a hearty supper, and was soon thereafter taken with violent retching and vomiting, and ejected all the ingesta he had taken for his dinner and supper. Pulse was 100, full, hard and regular; temperature subnormal; eyes turned toward the left side; pupils normal; was somewhat unconscious, but could be aroused so as to answer questions intelligently. No pain was complained of in the head; although temporal arteries were dilated with dark blood.

The patient was given full doses of calomel, bismuth, pepsin, and magnesia, ordered in the morning. Medicine acted freely on the bowels; eyes straight; pupils normal, responding to the action of light. Tongue protrudes straight. Pulse 110, full, hard and regular; temperature subnormal; nausea and vomiting continues; no increase of the old paralysis.

22d. Not so much vomiting; mind clear; no pain in the head; has that peculiar cephalic sigh so characteristic of diseases of the brain. Carbolic acid and lime water given up to the 25th, when the vomiting ceased.

On the 26th the pulse was found to be full, hard, and regular; temperature 1.2 subnormal; eyes straight; pupils normal and respond to the action of light; no pain in the head; patient is conscious and recognizes his friends when they call to see him; no increase of paralysis; he rolls himself in the bed from side to side and seems to use his left about as well as his right side. He has an occasional deep cephalic sigh. He has no thirst, but takes milk as food and drink. Prognosis unfavorable. Diagnosis uncertain.

On the 27th, Dr.———, an old friend of the family, was called in consultation and advised the use of Norwood's tincture of veratrum to diminish the frequency of the pulse and take off the blood pressure from the brain. Gave five drops every hour till the pulse was reduced to 80, then every two hours.

28th. No change in his general appearance; pulse 80; temperature $2\frac{1}{4}$ subnormal. No special nervous symptoms, such as twitching of the muscles.

30th. Pulse 100; temperature $1\frac{1}{2}$ subnormal; patient losing strength; is quite conscious; eyes normal; pupils respond to light; no pain; takes milk when it is offered to him; is restless and has had but little refreshing sleep. Cephalic sigh every fifteen or twenty minutes. The veratrum was given occasionally during the day and hydrate of chloral in ten-grain doses during night to procure sleep and quiet his restlessness. From this time up to the day of his death, there were no special symptoms other than those that have been given, except a gradual diminution of strength caused by some obscure disease of the brain.

Dr. Skeer desired to emphasize the condition of the patient on the 5th of October. He was conscious, pupils normal, and responded readily to the action of light. There was no increase of paralysis, the patient seeming to use his left as well as his right side. Muscular movements, however, were somewhat impaired. Death occurred on October 7th, at 7 p. m., apparently from exhaustion of the brain, with a group of symptoms that he could neither analyze nor formulate with any degree of certainty.

*Post-mortem examination.* There was nothing abnormal in the appearance of the upper surface of the brain. Some of the puncta vasculosa were very large; one small artery on the right side, when cut off on a line with the upper surface of the corpus callosum, stood open. There were about two drachms of serum in the ventricles and about four at the base of the brain. A blood cavity was found in the inner, upper, and posterior portion of the right hemisphere of the cerebellum, which was lined with a thin membrane and filled with serum and a clot of blood. The center of the right corpus was slightly softened. No tubercle, fibroma or carcinoma was found, nor even a trace of inflammatory action. The patient died from exhaustion of the brain, its nutrition having been cut off by general atheromatous degeneration of the cerebral arteries, which was the first link in the chain of morbid action, was the cause of the hæmorrhage and the softening of the corpus striatum, and readily accounts for the cerebral anæmia, the absence of pain in the head, and the subnormal temperature.

In reply to Dr. Angear, Dr. Skeer said the respirations were slow.

Dr. Angear: If we interfere with the action of the pneumogastric nerve we always increase the action of the heart, diminish the rate of respiration, and lower the temperature. If we have both an increased pulse and respiratory rate, and an increased temperature, all in due proportion, then we have no particular evidence of cerebral trouble. But if we get that "see-saw" arrangement, one going up while the other goes down, it is reasonably certain that there is interference with the pneumogastric nerve, and I think that in Dr. Skeer's case had the respirations been counted as accurately and persistently as that of the pulse, we should have found that they were far below their normal proportion.

